# *In Vivo* Confocal Microscopy and Anterior Segment Optic Coherence Tomography Findings in Ocular Ochronosis

**DOI:** 10.1155/2015/592847

**Published:** 2015-12-15

**Authors:** Elif Demirkilinc Biler, Suzan Guven Yilmaz, Melis Palamar, Pedram Hamrah, Afsun Sahin

**Affiliations:** ^1^Ege University Faculty of Medicine, Department of Ophthalmology, 35040 Izmir, Turkey; ^2^Department of Ophthalmology, Tufts Medical Center, Boston, MA, USA; ^3^Osmangazi University Faculty of Medicine, Department of Ophthalmology, Eskişehir, Turkey

## Abstract

*Purpose*. To report clinical and* in vivo* confocal microscopy (IVCM) findings of two patients with ocular ochronosis secondary due to alkaptonuria.* Materials and Methods*. Complete ophthalmologic examinations, including IVCM (HRT II/Rostock Cornea Module, Heidelberg, Germany), anterior segment optical coherence tomography (AS-OCT) (Topcon 3D spectral-domain OCT 2000, Topcon Medical Systems, Paramus, NJ, USA), corneal topography (Pentacam, OCULUS Optikgeräte GmbH, Wetzlar, Germany), and anterior segment photography, were performed.* Results*. Biomicroscopic examination showed bilateral darkly pigmented lesions of the nasal and temporal conjunctiva and episclera in both patients.* In vivo* confocal microscopy of the lesions revealed prominent degenerative changes, including vacuoles and fragmentation of collagen fibers in the affected conjunctival lamina propria and episclera. Hyperreflective pigment granules in different shapes were demonstrated in the substantia propria beneath the basement membrane. AS-OCT of Case 1 demonstrated hyporeflective areas. Fundus examination was within normal limits in both patients, except tilted optic discs with peripapillary atrophy in one of the patients. Corneal topography, thickness, and macular OCT were normal bilaterally in both cases.* Conclusion*. The degenerative and anatomic changes due to ochronotic pigment deposition in alkaptonuria can be demonstrated in detail with IVCM and AS-OCT. Confocal microscopic analysis in ocular ochronosis may serve as a useful adjunct in diagnosis and monitoring of the disease progression.

## 1. Introduction

Alkaptonuria (AKU) is a disorder of phenylalanine/tyrosine metabolism due to a defect in the enzyme homogentisate 1,2-dioxygenase (HGD). This recessive disease is caused by mutations in the HGD gene, leading to a build-up of homogentisic acid (HGA), a tyrosine degradation product, which can cause degenerative arthritis and calcific aortic stenosis [1]. Excess HGA is deposited as an ochre-colored pigment in collagen-rich tissues, such as bone, cartilage, and skin, causing a bluish-black discoloration, termed “ochronosis.” In “ocular ochronosis,” which is very rare, ochronotic pigmentation can be found in the sclera, conjunctiva, and limbic cornea as reported in single-case presentations [2–4]. Ochronotic pigments in the eye can be observed as bilateral, asymmetrical depositions in the interpalpebral fissure on slit-lamp biomicroscopy and may appear in different colors ranging from grey-brown to blue-black [5]. In a previously reported case, it has been shown that ochronotic pigmentation of the eye may cause progressive peripheral corneal thinning and results in the induction of astigmatism in the axis of the lesion [6].

*In vivo* confocal microscopy (IVCM) allows for real-time examination of the human cornea at the cellular level and provides coronal optical sections through the structure examined. Laser IVCM is a growing technique for the study of the cornea and conjunctiva at the cellular level and has been widely used to evaluate both their physiological and pathological states in the living eye [7–10].

The aim of this study is to report laser IVCM, corneal topography, and anterior segment optic coherence tomography (AS-OCT) findings as well as clinical features of two cases with ocular ochronosis due to alkaptonuria. Corneal involvement was revealed with IVCM in a previous case report recently [11]. However, to the best of our knowledge, this is the first report on the IVCM appearance of prominent scleroconjunctival involvement in concordance with AS-OCT findings.

## 2. Materials and Methods

Complete ophthalmologic examinations including corneal topography with a Pentacam rotating Scheimpflug camera (OCULUS Optikgeräte GmbH, Wetzlar, Germany), anterior segment photography (Topcon DC1 Digital Camera Unit, Topcon Medical Systems, Paramus, NJ, USA), AS-OCT (Topcon 3D spectral-domain OCT 2000, Topcon Medical Systems, Paramus, NJ, USA), and macular OCT (Topcon 3D spectral-domain OCT 2000, Topcon Medical Systems, Paramus, NJ, USA) were performed in two patients with ocular ochronosis. The patients were unrelated to each other. The laser IVCM images of the pigmented lesions were obtained using Heidelberg Retina Tomograph II with a Rostock Cornea Module (HRT II/RCM; Heidelberg Engineering GmbH, Heidelberg, Germany).

The HRT II/RCM is a contact confocal microscope constructed to examine the cornea* in vivo*. It operates with a 363 Zeiss objective, allowing a scanning area of 400 × 400 mm with a magnification up to 800 times and a resolution of approximately 1 *μ*m. The head of the patient must be positioned in the headrest and a fixation tool was used to perform confocal imaging of the structures. At least fifty images were taken during each examination (30 frames/sec) and stored for analysis. Before image acquisition one drop of proparacaine was instilled into inferior cul-de-sac. Pictures were recorded along the *z*-axis as section scans or as volume scans in the movie-motion mode. Scans were recorded from the nasal and/or temporal bulbar conjunctiva. The HRT II/RCM provides images measuring 400 × 400 *μ*m and the system can visualize the conjunctiva up to 200 *μ*m depth from the epithelial surface.

The local medical ethics committee approved the study. The research protocol adhered to the Declaration of Helsinki for research involving human subjects. The patients were informed of the procedures and their consent was obtained prospectively.

**Case 1 .**

**Figure 1:**
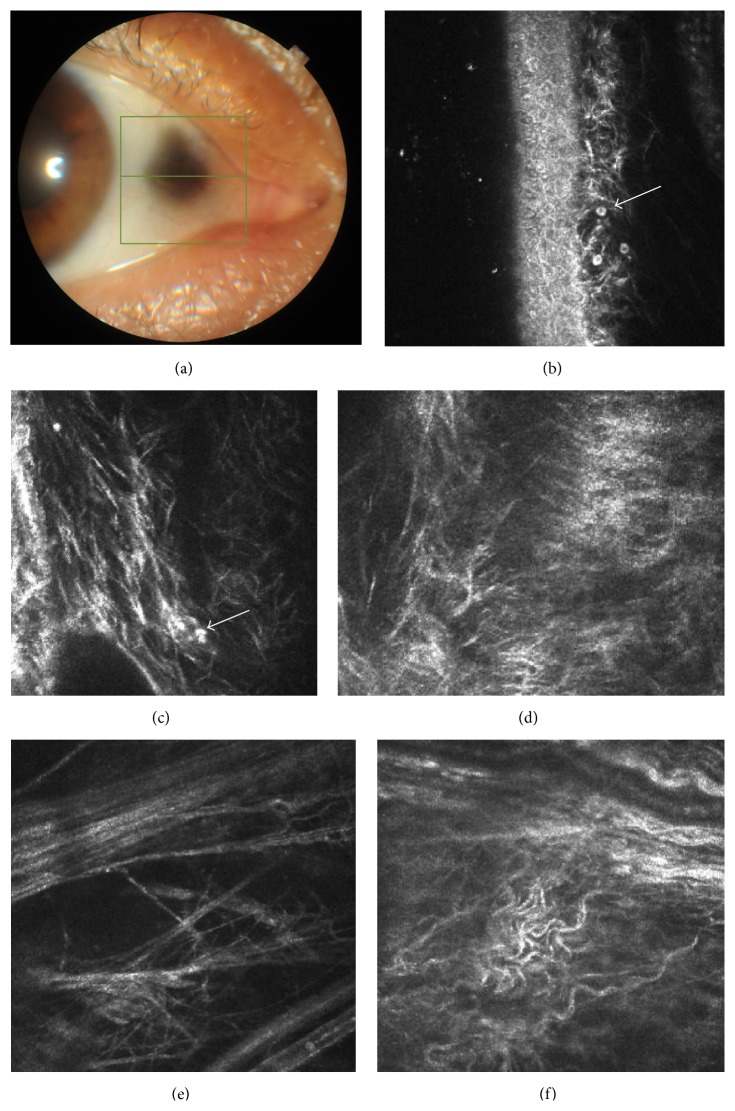
Anterior segment photos and* in vivo* confocal microscopy images of Case 1. (a) Right eye temporal conjunctiva. (b) Hyperreflective inflammatory cells beneath conjunctiva epithelium (arrow). (c) Hyperreflective deposits in the deep conjunctiva with adjacent curled collagen fibers (arrow). A normal lymphatic vessel is seen adjacent to curled collagen fibers. (d) Degenerative changes in the collagen. (e) Fragmentation of the collagen fibers and empty spaces between them. (f) Prominent curled subepithelial fibers in the deep conjunctiva. A normal lymphatic vessel is seen in the upper part of the scan.

**Figure 2:**
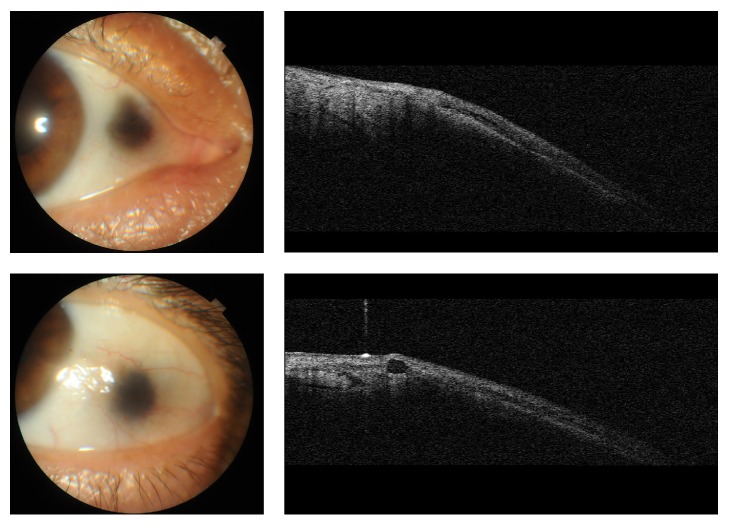
Anterior segment OCT of Case 1. Multiple hyporeflective areas with depth of 60 to 100 microns and width of 400 to 600 microns.

A 50-year-old male with the diagnosis of genetically confirmed (HGD gene c.175delA mutation) alkaptonuria was referred for dark pigmentation in both eyes. He had a past medical history of large-joint arthropathy and reported that since childhood his urine had turned into dark color upon exposure to air. He was also noted to have dark pigmentation of axillae and blue pigmentation of auricles. Elevated levels of homogentisic acid were found in his urine. He had +3.00 × 90 D astigmatism in the right eye and +4.00 × 80 D astigmatism in the left eye and his best-corrected visual acuity was 0.9 in the right eye and 0.8 in the left eye due to meridional amblyopia. On slit-lamp examination, bilateral asymmetrical dark pigmented lesions in both nasal and temporal interpalpebral conjunctiva and episclera were observed (Figure 1). The dimensions of the lesions in temporal and nasal conjunctivas were 3.2 × 3.2 mm and 3 × 3 mm in the right eye, respectively, and 2.8 × 2.8 mm and 1.5 × 1.8 mm in the left eye, respectively. In AS-OCT there were multiple hyporeflective areas with depth of 60 to 100 microns and width of 400 to 600 microns (Figure 2). No corneal involvement was detected in this patient. Posterior segment examination revealed tilted optic discs, peripapillary atrophy, and chorioretinal atrophy in both eyes. Central corneal thickness, which was measured by corneal topography, was 567 *μ*m in the right eye and 573 *μ*m in the left eye. Corneal topography (keratometric values are 42.25/40.75 diopters for right eyes and 42.75/40.25 diopters for left eyes) and macular OCT was bilaterally normal.

Laser IVCM of the pigmented lesions revealed prominent degenerative changes, including fragmentation of the collagen fibers and empty spaces between them in the affected conjunctival substantia propria. Hyperreflective pigment granules were shown in superficial stroma as round shaped aggregates or amorphous microdeposits (Figure 1).

**Case 2 .**

**Figure 3:**
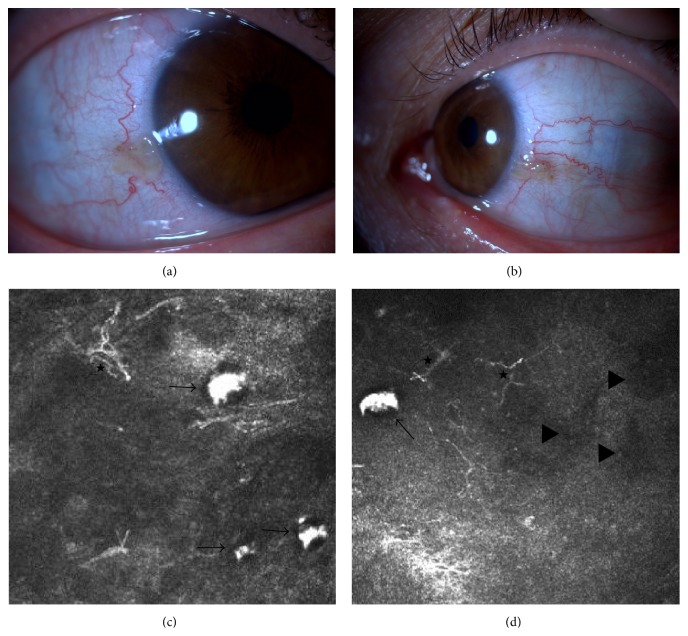
Anterior segment photos and* in vivo* confocal microscopic images of Case 2, 400 × 400 *μ*m. (a) Right eye temporal conjunctiva. (b) Left eye temporal conjunctiva. (c) Hyperreflective deposits presumed to be homogentisic acid accumulation beneath conjunctival epithelium (black arrows) and dendritic cells (star). (d) Hyperreflective homogentisic acid deposit (arrow) and hyporeflective areas (arrow head) in the conjunctiva close to limbus.

A 59-year-old female with alkaptonuria was referred for blue-black pigmentation in both eyes. She has been complaining of dark coloration of her urine for 20 years and has also noted dark pigmentation of her axillary region. Elevated levels of homogentisic acid were detected in her urine. She was diagnosed with alkaptonuria and has been followed by the rheumatology department for the past 10 years for spondyloarthropathy and scoliosis. Her visual acuity was 1.0 in both eyes. On ophthalmologic examination, bilateral asymmetrical dark pigmented lesions were observed in both the nasal and temporal interpalpebral conjunctiva. Areas of conjunctival degeneration and deep sclerocornea involvement in bilateral temporal limbus were detected (Figures 3(a) and 3(b)). Additional pigmented microlesions were also observed in the limbal area in both eyes. The dimensions of the lesions in temporal and nasal conjunctiva were 2.0 × 1.8 mm and 3 × 3 mm, respectively, in the right eye and 2.8 × 1.8 mm and 2.2 × 2.5 mm in the left eye, respectively. Posterior segment examination revealed no pathologic findings. Corneal topography (keratometric values are 47.75/48.75 diopters for right eyes and 47.00/46.00 diopters for left eyes) and macular OCT were normal bilaterally.

Laser IVCM of the pigmented conjunctival lesions demonstrated epithelial and subepithelial hyperreflective deposits with jagged borders, often forming crescent or banana shapes with the vacuolar degeneration around the aggregates. The lesions were surrounded by dendritiform cells and were found to be mostly located in the stroma (Figures 3(c) and 3(d)).

## 3. Discussion

In AKU, the most serious consequence of the excessive HGA is the accumulation of ochronotic pigment in collagenous tissues, such as cartilage and tendon, especially in the joints, disc spaces, ear cartilage, heart valves, nose, nails, cheeks, and ocular tissues [2, 4]. Although the metabolic defect is present from birth, degenerative arthropathy, spondyloarthropathy, and cardiovascular disease develop slowly and are usually clinically evident by the fourth decade of life [1]. Ocular ochronosis more frequently involves sclera and episclera near the insertion of the recti muscle (interpalpebral areas) than in the cornea and conjunctiva [2, 12]. Often, ocular pigmentation is one of the initial manifestations of the disease [4]. Although conjunctival involvement in ochronosis is rare, it should be considered in the differential diagnosis of pigmented lesions and deposits of the ocular surface, such as extraocular extension of intraocular malignant melanoma [4, 12]. It has been postulated that perilimbal and scleral ochronotic lesions in the nasal and temporal eye regions may result in progressive peripheral corneal thinning and astigmatism in the axis of lesion [6]. However, no corneal thinning or prominent astigmatism was detected in any of our patients.

Corneal pigmentation in ocular ochronosis is usually bilateral, asymmetric and presents in the peripheral stroma as discrete pinhead sized deposits of light brown to black color [2, 4]. Carlson et al. reported the accumulation of amber-colored pigmented lesions in Bowman's layer of the cornea [2]. This ochronotic pigment was demonstrated on light microscopy by Kampik et al. as amber globules or fiber-like structures in the cornea, conjunctiva, and sclera combined with degenerated collagen fibrils [13]. Although the ultrastructure of the ochronotic pigment was similar to melanin, the chemical behavior was different, and it seemed to be similar to elastin. Ultrastructurally, most of the pigment granules were extracellular, partly altering the collagen fibers and fibrocytes [4, 13]. In a recent study, accumulation of hyperreflective crystalline deposits at the level of Descemet membrane and scattered hyperreflective microdeposits more superficially was shown via IVCM in a case of ocular ochronosis with corneal involvement [11].

Histopathologic examination shows globules or curled, light yellow, curvilinear structures of varying size in the superficial stroma and surrounding tissues [4, 6]. Some authors have noted that areas of intense scleral pigmentation are devoid of cells, suggesting a probable toxic effect of the pigment [13]. Strikingly, we observed this finding in our case (Case 1). There are no cells in pigmented areas, supporting this toxic effect theory. It proposed a sequence, in which deposition of HGA polymers occurs in a fine granular form around collagen fibrils, altering and obscuring their structure. The granules later coalesce to form plaques, globules, and fiber-like structures, followed by necrosis of the fibrocytes [13]. Similar to their histological data, our IVCM results demonstrate hyperreflective amorphous microdeposits or deposits with jagged borders, forming round, crescent, or banana shapes, with accompanying degenerative changes and immune cells around these aggregates. It was proposed that these immune cells could be melanophages [14]. In our study, the lesions were surrounded mostly by dendritiform cells. Probably the changes differ due to the duration of the clinical manifestations and severity of the disease. Moreover, degenerative changes such as elastotic degeneration of collagen fibers are common in the limbal/close limbal area. Therefore, one should keep this in his/her mind while scanning areas close to limbus.

In conclusion, we demonstrated* in vivo* findings of ocular ochronosis with prominent conjunctival involvement by IVCM and AS-OCT. IVCM allows detailed examination of degenerative changes due to ochronotic pigment deposition in real-time and longitudinally. Since it is a rapid and noninvasive method, confocal microscopic analysis in ocular ochronosis may serve as a useful adjunct in diagnosis and especially monitoring the progression of the disease.
